# Osteogenic differentiation of periodontal membrane stem cells in inflammatory environments

**DOI:** 10.1515/biol-2022-0474

**Published:** 2022-09-19

**Authors:** Shenghao Jin, Haitao Jiang, Yue Sun, Fang Li, Jianglan Xia, Yaxin Li, Jiwei Zheng, Ying Qin

**Affiliations:** Department of Periodontics, School of Stomatology, Xuzhou Medical University, Xuzhou, Jiangsu, 221000, China; Department of Oral and Maxillofacial Surgery, Affiliated Hospital of Xuzhou Medical University, Xuzhou, Jiangsu, 221000, China

**Keywords:** periodontal membrane stem cells, osteogenic differentiation, inflammatory environment, lipopolysaccharide

## Abstract

Periodontitis is a common disease that is difficult to treat, and if not controlled in time, it causes severe conditions, such as alveolar bone resorption and tooth loosening and loss. Periodontal ligament stem cells constitute a promising cell source for regenerative treatment of periodontitis due to their high osteogenic differentiation capacity. PDLSC osteogenesis plays a central role in periodontal regeneration through successive cytokine-mediated signaling pathways and various biochemical and physicochemical factors. However, this process is inhibited in the inflammatory periodontitis environment due to high concentrations of lipopolysaccharide. Here, we review the mechanisms that influence the osteogenic differentiation of periodontal stem cells in this inflammatory microenvironment.

Periodontitis is an infectious disorder that occurs in the supporting tissues of the teeth and, if left untreated, often results in loosening and loss of teeth and jawbone loss. The commonly used clinical treatments, such as physiotherapy and periodontal surgery, do not yield favorable results. Therefore, tissue regeneration and the use of stem cells to reconstruct periodontal and bone tissues have recently become a major research topic. In recent years, with the development of the field of oral tissue regeneration, the discovery of oral-derived seed cells has enabled the complete regeneration of the periodontal tissue. The cells [1] that have been found to play a regenerative role are partially used in clinical applications include dental pulp stem cells, stem cells from the apical papilla, dental follicle progenitor cells, and periodontal ligament stem cells (PDLSCs). Many studies [2,3,4] have shown that odontogenic stem cells are superior to non-dental stem cells in regenerative treatment, and periodontal stem cells are recognized as one of the safest and most effective stem cells for this process. PDLSCs are mesenchymal stem cells with self-renewal and multi-directional differentiation potentials (Figure 1), whereby they can differentiate into various types of mesenchymal cells [5], such as osteoblasts, adipocytes, and chondrocytes, in addition to cardiomyocytes, endothelial cells, and ectoderm-derived neural cells, namely neurons, oligodendrocytes, astrocytes, and Schwann cells. Thus, PDLSCs constitute a promising cell source for the repair of various types of tissues in regenerative medicine. Liu et al. [6] and Nagata et al. [7] have investigated the role of PDLSCs in the repair of periodontal bone defects and demonstrated that PDLSC-derived cells are highly effective in osteogenesis and in the formation of a periodontal-like tissue, whereby the regeneration and repair of the periodontal attachment tissue are promoted. These studies have laid the foundation for the development of regenerative treatment approaches in periodontitis.

**Figure 1:**
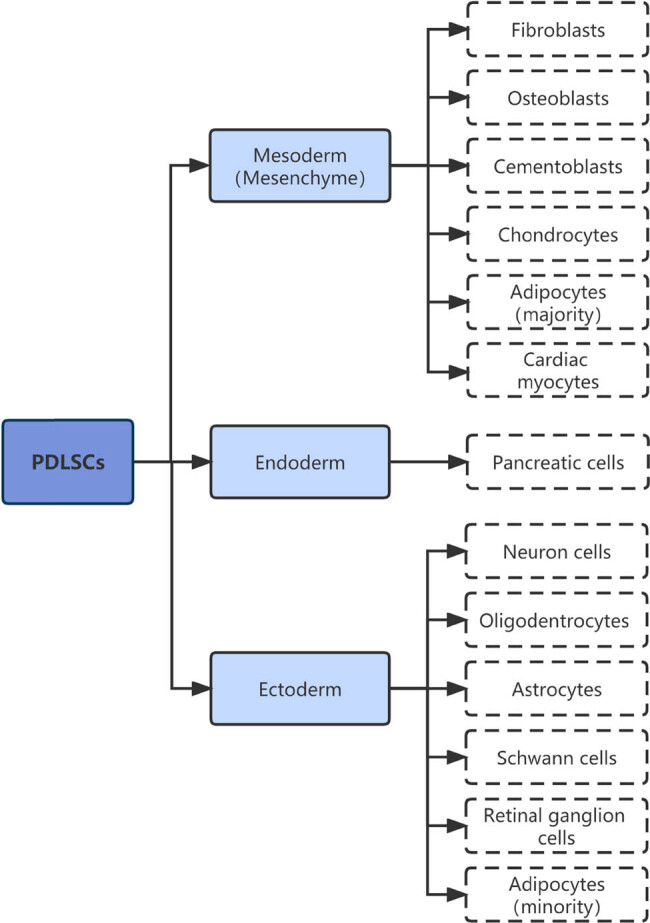
Multi-directional differentiation potential of periodontal stem cells. (Description: This figure describes the multidirectional differentiation potential of PDLSCs. According to the sources of endoderm, mesoderm, and ectoderm, PDLSCs can differentiate into a variety of cells in the figure. We can use this feature to study its role in repairing bone and other tissue defects in the treatment of periodontitis.).

The bacteria [8] that presumably cause periodontitis include 11 species, such as *Actinobacillus*, *Actinomyces*, *Porphyromonas gingivalis*, and *Fosetanella*, most of which are G-bacteria. Lipopolysaccharide (LPS) is an important component of the G-bacterial membrane, and the LPS-induced inflammatory microenvironment is an important and typical manifestation of periodontitis. This inflammatory microenvironment induces production of numerous inflammatory factors, such as TNF-ɑ, IL-6, and IL-1β, and involves various signaling pathways. According to the recent advances in periodontal disease microbiology, there is conclusive evidence [9] that some of these bacteria, such as *P. gingivalis* and *Bacillus actinomycetemcomitans*, play important roles in the pathogenesis of periodontitis, including the activation of the inflammatory response and promotion of bone loss, both of which are inextricably linked to the production of LPS by bacteria. Although the effects of different bacterial species on periodontal regeneration after bone loss are unclear, the effect of LPS on the osteogenic differentiation of stem cells has commonalities. Croes et al. have found [10] that osteogenesis is active after stimulation with small amounts of inactivated bacteria (low LPS levels). Based on this observation, numerous studies [11,12,13] have confirmed the protective, proliferative, and osteogenic effects of low concentrations of LPS (generally <5 μg/mL) on periodontal cells. The underlying mechanism may stem from an adaptive mechanism of PDLSCs at low concentrations of LPS. Alternatively, low concentrations of LPS may induce PDLSCs to activate the macrophage NRF2 signaling pathway to regulate the oxidative stress and downregulate macrophage pro-inflammatory factor expression. However, this is obviously not consistent with the microenvironment of severe periodontitis, and thus, high LPS concentrations should be considered in studies. At high LPS concentrations [14], cell proliferation and osteogenic differentiation are significantly reduced, presumably due to the induction of apoptosis through the classical NF-kB pathway and the action of cystein. Studies have generally explored LPS concentration gradients to identify the mechanism(s) underlying these two opposing effects of LPS. However, Albiero et al. [15] have observed that the osteogenic potential of LPS on periodontal stem cells is not affected. This observation has encouraged researchers to seek substances that favor the osteogenic differentiation of stem cells in the periodontitis environment, with the ultimate aim to improve the efficacy of current therapies and to find new therapeutic strategies. The following is a summary of the various mechanisms and factors influencing the osteogenic differentiation of PDLSCs under LPS-induced inflammatory conditions.

## 1 Roles of major signaling pathways

### 1.1 Wnt/β-catenin signaling pathway

**Figure 2:**
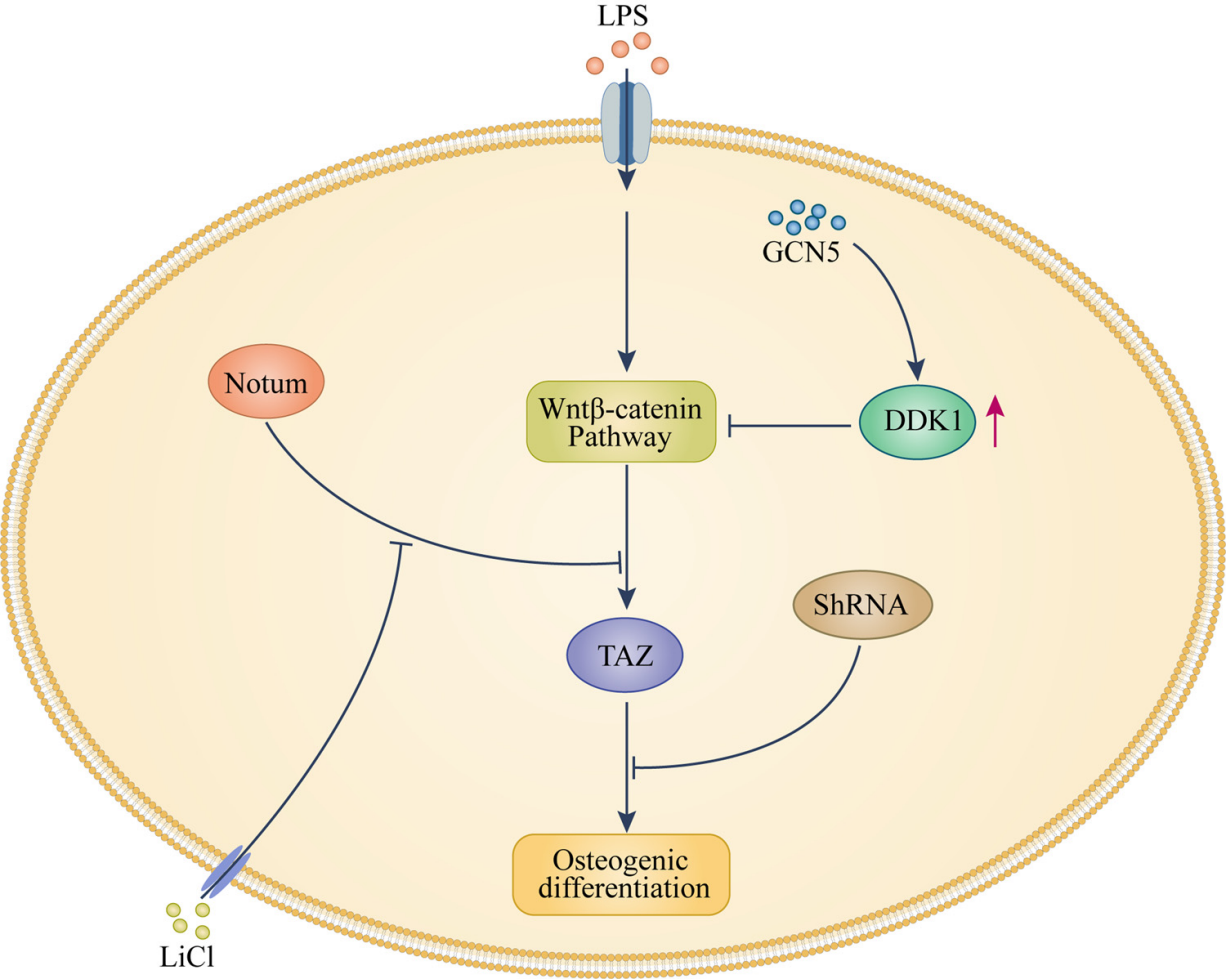
Mechanism of Wnt/β-catenin signaling pathway (Description: Wnt/β-catenin pathway is important for osteogenic differentiation of PDLSCs. In the presence of LPS, histone acetyltransferase GCN5 can inhibit this pathway through the acetylation regulation of DDK1. TAZ, which is necessary for LPS-induced osteogenesis, is a downstream component of this signal pathway. Interference with it by ShRNA or under the influence of phospholipase notum can inhibit Wnt/β-catenin pathway-mediated osteogenic differentiation of PDLSCs, and LiCl could reverse the effect of Notum).

Transcriptional co-activator with PDZ binding motif (TAZ, also known as WWTR1) is a transcriptional co-regulator that promotes the osteogenic differentiation of PDLSCs. Xing et al. [16] observed that LPS-induced osteogenic differentiation of PDLSCs is suppressed upon knocking down TAZ, indicating that TAZ is required for LPS-induced osteogenesis. They also found that the use of the Wnt/β-catenin pathway inhibitor DKK inhibits LPS-induced TAZ activation and osteogenesis, indirectly confirming that TAZ is a downstream component of the Wnt signaling pathway, thus inferring that low concentrations of *Escherichia coli* LPS promote the osteogenic differentiation of human PDLSCs (hPDLSCs) through Wnt/ntcatenin-induced TAZ upregulation. This result is encouraging as it explains to some extent the osteogenic effect of LPS at low concentrations. Furthermore, Li et al. [17] found that, in PDLSCs, removal of the histone acetyltransferase GCN5 downregulates DKK1, whereby the Wnt/β-catenin pathway is activated and osteogenesis is promoted. Thus, it is reasonable to infer that GCN5 regulates the osteogenic differentiation of periodontal stem cells in an inflammatory microenvironment through DKK1 acetylation (Figure 2).

Recently, Notum, a glycoprotein glycan and Wnt/β-catenin-associated ligand, has been discovered. It is a phospholipase shed on the cell surface. Yang et al. [18] examined the expression of Notum in LPS-treated PDLSCs and the osteogenic markers under the influence of Notum. By inhibiting the effect of Notum on the Wnt/β-catenin pathway, they found that this protein may inhibit the osteogenic differentiation of hPDLSCs through this pathway. In addition, lithium chloride [19,20] significantly suppressed the inhibitory effect of Notum on the osteogenic differentiation of hPDLSCs. These observations provide a new thinking for the treatment of periodontitis.

### 1.2 Toll-like receptor TLR 4/nuclear factor NF-κB signaling pathway

**Figure 3:**
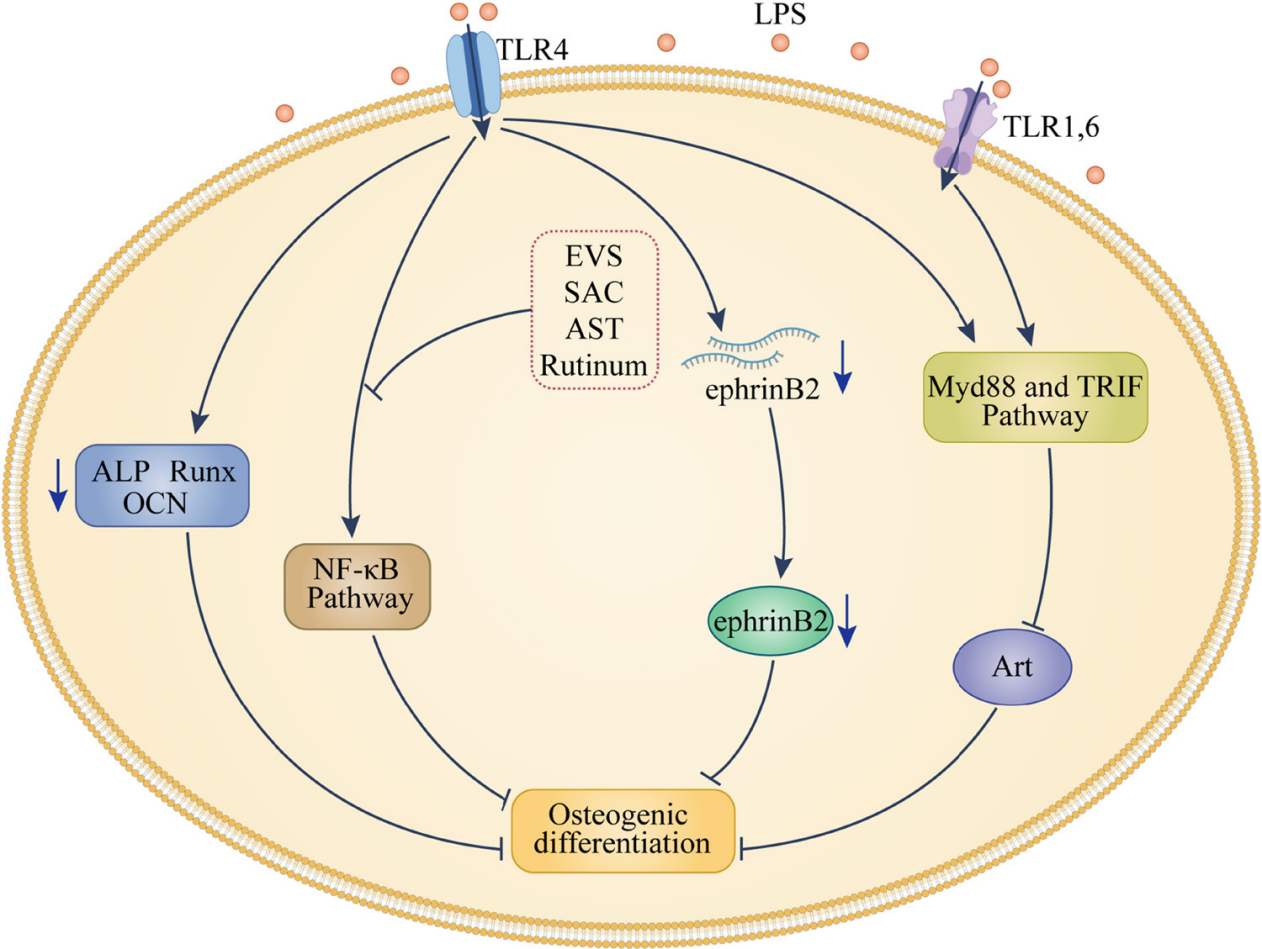
Mechanism of Toll-like receptor TLR 4/nuclear factor NF-κB signaling pathway (Description: transmembrane receptor TLR4 can participate in the activation of signal pathways under inflammatory conditions. In LPS-mediated environment, TLR4 receptor activation decreased the expression of osteogenic markers, such as ALP, Runx, and OCN, activated NF-kB pathway, and downregulated the mRNA expression of osteogenic marker EphrinB2. All these make the osteogenic differentiation of PDLSCs inhibited. The activation of TLR1,4,6 can inhibit the activation of Akt and osteogenic differentiation of PDLSCs through Myd88- or TRIF-dependent signaling pathways. In addition, some studies have found that the inhibition of NF-kb pathway can be reversed by rutin, extracellular vesicles or astaxanthin).

TLR4 is a transmembrane receptor involved in the activation of signaling pathways under inflammatory conditions. Yu et al. [21] applied TLR 4 agonists or antagonists to hPDLSCs extracted from patients with periodontitis alongside those from healthy individuals and analyzed indicators of cell proliferation and differentiation. The authors found that TLR4 activation decreases the ALP activity and expression of osteogenic markers but increases the expression of adipogenesis-related genes poly(AdP ribose) polymerase gamma and lipoprotein lipase, demonstrating that LPS can inhibit the osteogenic differentiation of hPDLSCs and induce their adipogenic differentiation under inflammatory conditions by activating the TLR4 signaling (Figure 3).

The TLR4 receptor-mediated signaling is common, and it is also closely associated with other inflammatory pathways. By using anti-TLR4 antibodies or antagonizers of the TLR4 or NF-κB signaling, Li et al. [14] found that LPS reduces the osteogenic differentiation of hPDLSCs by activating NF-κB through the TLR4 signaling pathway. The LPS-induced reduction in osteogenesis suppressed the alveolar bone loss in LPS-induced periodontitis in rats. Furthermore, Duan et al. [22] observed that salvianolic acid C promoted osteogenesis by attenuating LPS-induced inflammation and apoptosis through the inhibition of the TLR4/NF-κB pathway.

Many factors affect the TLR4 signaling pathway. Wang et al. [23] found that various concentrations of LPS downregulated the mRNA level of *EphrinB2*, a cellular marker of osteodifferentiation. In contrast, blocking the TLR4 pathway partially suppressed this effect of LPS. Therefore, it is inferred that LPS partially inhibits the osteogenesis of hPDLSCs by downregulating EphrinB2 through the TLR4 signaling.

In addition to TLR 4 receptor, experiments on other receptors of the same type confirmed that a common signaling feature of all TLRs is the activation of the transcription factor NF-κB, which controls the expression of inflammatory cytokines and factors involved in cell maturation. Through analyses based on polymerase chain reaction, Zhu et al. [24] found that high doses of TLR1, 4, and 6 ligands inhibit the expression of NF-κB. This inhibition occurs through Myd88 or the TRIF-dependent signaling pathway to inhibit Akt activation and hPDLSC osteogenic differentiation in turn. In addition, it has been shown that rutin [25] and extracellular vesicles [26] or astaxanthin [27] can increase the anti-oxidative stress capacity, proliferation, and osteogenic differentiation of PDLSCs by inhibiting the activity of the NFκB signaling, all of which lay the foundation for the development of new treatment strategies in periodontitis.

### 1.3 ERK1/2 signaling pathways [28]

It has been shown that activation of the ERK1/2 signaling pathways by LPS is at least partially involved in the alteration in the differentiation capacity of PDLSCs as well as in the acquisition of myofibroblast and immunoregulatory properties of these cells. LPS downregulates regulatory genes related to osteogenesis, such as *Runx2*, *ALP*, and *Ocn*, thereby altering the differentiation commitment of PDLSCs and acting as a suppressor of osteogenesis. In addition, LPS upregulates *Sox9* and *PPARγ*, which promote chondrogenesis and adipogenesis. PDLSC-derived myofibroblasts acquire significant contractile motility upon activation of the ERK1/2 signaling, indicating the effect of this signaling pathway in cell differentiation.

## 2 Effect of microRNAs (miRs) and IncRNAs

miRs are a class of non-coding single-stranded RNAs that are approximately 22 nucleotides in length. They are encoded by endogenous genes and involved in post-transcriptional regulation in plants and animals. Bao et al. [29] found that *miR-148a* is upregulated whereas neurofibrillary protein1 (NRP1) is downregulated when LPS inhibits PDLSC proliferation. Conversely, downregulation of *miR-148a* or upregulation of NRP1 increased the osteogenic capacity of LPS-stimulated PDLSCs. Inhibition of NRP1 could negate the promotion of osteogenesis by *miR-148a* inhibitors. These two actions are complementary and not independent, confirming the osteogenic potential of PDLSCs in an LPS-induced inflammatory environment. The decrease in the osteogenic potential of PDLSCs in such an environment is associated with the *miR-148a*/NRP1 functional axis (Table 1).

**Table 1:** Effect of miRs and IncRNAs and other genes (description: the table lists some genes that affect the osteogenic differentiation of periodontal stem cells and their mechanisms, especially miRNA and IncRNA)

Gene	Mechanism	Effect on osteogenesis
*miR-148a/NRP1 functional axis* [29]	Downregulating *miR-148a* or upregulatiing of NRP1	Promoting the osteogenic capacity of LPS-stimulated PDLSCs
*miR-21* [30]	Inhibiting a negative regulator (Spry1) of the ERK and FGF signaling pathways	Promoting lipogenesis and osteogenesis of PDLSCs
*miR-200cN* [31]	Inhibiting LPS-induced IL-6, IL-8, and CCL-5 production in hPDLSCs and increasing cellular calcium, *ALP*, and *Runx2* levels	Promoting osteogenic differentiation
*miR-138* [31]	Downregulating osteocalcin, *Runx2*, and type I collagen	Inhibiting the osteogenic differentiation of PDLSCs
*miR-26a-5p* [32]	Presumably targeting Wnt5a and thereby inhibiting the activation of the Wnt/Ca^2+^ signaling	Promoting the osteogenic differentiation of PDLSCs
*miR-302b* [37]	Using Chuanxiongzin (TMP) to reduce inflammation and apoptosis	Promoting osteogenesis of LPS-stimulated human periodontal membrane cells
*IncRNA* [36]	Presumably regulating the metabolism of various amino acids	Promoting the osteogenic differentiation of PDLSCs
*P2X7R* [33,34]	Affecting the PI3K/Akt/mTOR signaling	Promoting the osteogenic differentiation of PDLSCs
*HOXA10* [35]	Regulating β-linked protein localization and DKK1	Inhibiting the osteogenic differentiation of periodontal stem cells
*PLAP-1* [38]	Using 1,25(OH)_2_D_3_ to inhibit the transcription of *PLAP-1*	Promoting osteogenesis

Yang et al. [30] found that *miR-21* can promote the lipogenesis and osteogenesis of PDLSCs by directly inhibiting *Spry1*, a negative regulator of the ERK and FGF signaling pathways. However, TNF-α may control the expression of inflammatory cytokines, adipogenesis, and osteogenic differentiation of PDLSCs by targeting the *miR-21/Spry1* functional axis.

In addition, several studies have identified other miRs that affect the osteogenic differentiation of PDLSCs. For instance, *miR-200cN* [31] inhibits LPS-induced IL-6, IL-8, and CCL-5 production in hPDLSCs and increases cellular calcium, *ALP*, and *Runx2* levels, consequently promoting osteogenic differentiation. Another miRNA, *miR-138*, hinders the osteogenic differentiation of PDLSCs by downregulating osteocalcin, *Runx2*, and type I collagen. Conversely, *miR-26a-5p* [32] promotes the osteogenic differentiation of PDLSCs both *in vitro* and *in vivo*, presumably by targeting Wnt5a and thereby inhibiting the activation of the Wnt/Ca^2+^ signaling. Xu et al. [33,34] found that the inflammation-mediated changes in *P2X7R*-overexpressing PDLSCs in an inflammatory osteogenic microenvironment were significantly reduced. Another study revealed that *P2X7R* overexpression could significantly enhance the osteogenic differentiation of PDLSCs through the *PI3K/Akt/mTOR* signaling. These two studies paved the way for the gene modification of stem cells for the treatment of periodontitis. miR species and effects are far from that. Recently, Wang et al. [35] demonstrated experimentally that *HOXA10* inhibits the osteogenic differentiation of periodontal stem cells through the regulation of β-linked protein localization and DKK1.

lncRNA is a non-coding RNA of >200 nucleotides. Based on the known IncRNAs, Zhang’s team [36] predicted 63 new lncRNAs that regulate the metabolism of various amino acids. They demonstrated that these metabolic pathways are important for the osteogenic differentiation of PDLSCs, thereby largely unraveling the mechanism underlying lncRNA regulation of osteogenesis. In addition, with the ultimate aim to improve the differentiation potential of periodontitis-derived PDLSCs, the same authors pursued the mechanism, whereby an inflammatory microenvironment hinders osteogenesis and consequently identified 318 differentially expressed lncRNAs between healthy PDLSCs and LPS-mediated PDLSCs. The list of these lncRNAs is expected to be a useful tool to study the osteogenic differentiation of PDLSCs under inflammatory microenvironment. These lncRNAs are expected to be valuable in studying the mechanisms underlying the osteogenic differentiation of PDLSCs in an inflammatory microenvironment. Such studies on the mechanisms whereby lncRNAs regulate the osteogenic differentiation of PDLSCs have recently flourished.

In addition, several scholars have explored chemical approaches to improve osteogenesis. For example, tetramethylpyrazine (TMP) [37] improved the osteogenesis of LPS-stimulated human periodontal membrane cells by reducing inflammation and apoptosis through downregulation of *miR-302b*. In addition, 1,25(OH)_2_D_3_ has been shown to promote osteogenesis by inhibiting the transcription of *PLAP-1* [38], but clinical trials have not yielded promising results.

## 3 Effects of biochemical and other mechanisms

Epigenetic modifications, such as DNA methylation and histone acetylation, manifest at the molecular level. Diomede et al. [39] detected that protein levels are altered in association with DNA methylation and histone acetylation in LPS-treated hPDLSCs and inferred that LPS can affect the osteogenic differentiation of periodontal stem cells through epigenetic modifications.

Endoplasmic reticulum stress (ERS) is a pathophysiological process [40,41] in which an imbalance in the action of the endoplasmic reticulum in response to a noxious stimulus leads to a decrease in the ability of the endoplasmic reticulum to fold proteins and an increase in the amount of unfolded proteins. Xue et al. [42] applied endoplasmic reticulum activators to LPS-treated PDLSCs and detected significant downregulation of the osteogenic markers *RUNX2* and *ALP*. Zhang et al. [43] found that inflammatory periodontal stem cells mediated IL-1β secretion by macrophages through regulation of macrophage endoplasmic reticulum stress, which is suggestive of the involvement of endoplasmic reticulum in osteogenesis. Zhai et al. [44] found that upregulation of mitochondrial fusion protein 2 (mitofusin 2, Mfn2), a factor mediating endoplasmic reticulum-mitochondria coupling, suppresses osteogenesis. Recently, Feng et al. have found that 4-phenylbutyric acid [45] can inhibit inflammation and improve osteogenesis through ERS-related mechanisms and the NF-κB pathway.

From a systemic perspective, Plemmenos and Piperi [46] found that AGEs, glycosylation end products in diabetic metabolism, enhance LPS-induced expression of inflammatory factors in hPDLSCs and reduce osteogenic differentiation. It is suggested that diabetes may exacerbate the inflammation in patients with periodontitis and thus must be considered in periodontal treatment.

## 4 Conclusion

Periodontitis is a common disease of the periodontal supporting tissues, and most patients present to the clinic with severe symptoms, such as significant alveolar bone resorption and tooth loosening or even loss. However, there is currently no effective therapeutic strategy in periodontitis. In recent years, the development of tissue stem cell engineering has led researchers to seek breakthroughs in periodontitis treatment from a new perspective, such as regenerative approaches using periodontal stem cells. However, in the oral cavity of patients with severe periodontitis, the inflammatory microenvironment [47] caused by the high concentrations of LPS hampers the osteogenic differentiation of periodontal stem cells and, thus, impedes the success of the regenerative therapy. Therefore, the mechanisms underlying this phenomenon should be elucidated to find effective solutions that can improve the success rate.

PDLSC osteogenesis is differentially affected by LPS through different signaling pathways. The osteogenic differentiation of hPDLSCs is promoted by *E. coli* LPS at low concentrations through the Wnt/β-catenin–induced upregulation of TAZ. However, both the histone acetyltransferase GCN5 and phospholipase Notum can suppress this pathway, thereby inhibiting the osteogenic differentiation. LPS can also inhibit osteogenesis and instead induce adipogenesis of hPDLSCs under inflammatory conditions by activating the TLR4 signaling and presumably through the resulting *EphrinB2* downregulation, and by activating the NF-κB pathway. In addition, high doses of TLR1, 4, and 6 ligands inhibit Akt activation through the Myd88- or TRIF-dependent signaling pathways, which also inhibit hPDLSC osteogenesis. Activation of the ERK1/2 signaling can also affect cell differentiation. Studies at the gene level have revealed that the miR-148a/NRP1 functional axis and *miR-138* have inhibitory effects on osteogenesis, whereas *miR-200cN*, *miR-26a-5p*, and *P2X7R* have stimulatory effects. Studies on micro-RNAs and lncRNAs have expanded the spectrum of mechanistic studies related to osteogenesis. In addition, epigenetic modifications, ERS, endoplasmic reticulum-mitochondrial coupling, and diabetic metabolites have inhibitory effects on the osteogenic differentiation of periodontal stem cells.

Immense progress [48] has been made on the mechanisms affecting the osteogenic differentiation of PDLSCs in an LPS-induced inflammatory environment. These stem cells constitute a physiologically suitable cell population that can be used for the regenerative treatment of periodontitis [49]. Furthermore, they can be easily obtained and cultured and are tolerant to the LPS-induced inflammatory microenvironment of periodontitis. Of course, there are still many mechanisms yet to be clarified. First, the connections between the signaling pathways should be studied to further investigate the mechanisms affecting the osteogenic differentiation of PDLSCs in an LPS-induced inflammatory environment. Second, PDLSCs should be compared with other stem cells in osteogenic capacity in such an environment. Finally, although many factors affecting osteogenesis have been clinically explored, only a few have been applied to clinical experiments, and thus, basic research findings should be evaluated through additional clinical studies for bench-to-bedside translation.
